# Rheumatoid Arthritis in Childhood

**Published:** 1906-02-24

**Authors:** 


### RHEUMATOID ARTHRITIS IN CHILDHOOD.

Probably in no department of medicine does more confusion exist than in the conception of the disease or diseases embraced by the term "Rheumatoid arthritis." None the less the type is distinct enough. Rheumatoid arthritis, osteo-arthritis, arthritis deformans, rheumatic gout, all these names apply to a disease having a definite morbid representation—namely, a fibrillation and subsequent erosion of articular cartilage, a thickening of synovial membrane and the formation of osteophytes about the joint affected. Articular destruction and osteophyte-formation are of the essence of this malady, and no chronic joint affection should come under the category of rheumatoid arthritis or its synonyms in the absence of these two cardinal factors. Rheumatoid arthritis is a disease of middle and later life, but it may occasionally be met with in childhood, and in a typical form. But in the early years of life the disease which most often masquerades as rheumatoid arthritis is one quite distinctly marked out from the type of the latter disease. This disease of childhood was first differentiated by Still. It is characterised by a multi-articular arthritis of a sub-acute kind, often associated with fever. The disease almost always makes its appearance before the second dentition, and has as a notable association an enlargement of the lymphatic glands all over the body, and of the spleen. The enlargement of the joints is due to a general thickening of the circum-articular tissues, grating of the joint and osteophyte-formation being absent.

Yet a third variety of multiple arthritis in childhood is a rare form, sometimes seen in those of syphilitic inheritance. A case purporting to be of this nature has recently been recorded by Dr. Findlay and Mr. Riddell [1] under the title of "A gummatous synovitis of many joints closely simulating rheumatoid arthritis in a congenitally syphilitic child." The details of the case are as follows. The child was nine years old, the fourth child in a family of six, and the only tainted one as far as was known. This child had suffered from "snuffles" in infancy, had undergone an interstitial keratitis, and presented Hutchinson's teeth in a well marked degree. The history stated that in the seventh year of age the joints began to swell, the right knee being the first affected. Gradually the involvement of the joints had proceeded, until at the date of examination practically every joint in the body was affected. The glands in the neck and axillæ were slightly enlarged. The lesion of the joints appeared to be in the main a synovial one, and there was no formation of osteophytes.

The authors base their diagnosis, in the first place, upon the amelioration of the symptoms which followed the administration of mercury with potassium iodide, and upon the fact that the child was seven years old at the commencement of the symptoms, older, that is to say, by a year than the common limit of onset in the case of the arthritis of Still. In addition the cervical spine was not attacked, and the joints were painful, circumstances contrary to the rule in the case of Still's arthritis. On the facts presented it cannot be said that the authors have established their case. Although it may be admitted that there is a multiple arthritis associated with congenital syphilis, it is extremely rare, and the items of correspondence between the authors' case and the much more common disease described by Still leave upon the mind an impression that the syphilitic inheritance was a complicating, and perhaps an aggravating, factor, but nothing more.

[1] Glasgow Med. Jour., Jan. 1906.

